# Milligram Quantities of Homogeneous Recombinant Full-Length Mouse Munc18c from *Escherichia coli* Cultures

**DOI:** 10.1371/journal.pone.0083499

**Published:** 2013-12-31

**Authors:** Asma Rehman, Russell J. Jarrott, Andrew E. Whitten, Gordon J. King, Shu-Hong Hu, Michelle P. Christie, Brett M. Collins, Jennifer L. Martin

**Affiliations:** 1 Division of Chemistry and Structural Biology, University of Queensland, Institute for Molecular Bioscience, Brisbane, Australia; 2 Division of Molecular Cell Biology, University of Queensland, Institute for Molecular Bioscience, Brisbane, Australia; Aligarh Muslim University, India

## Abstract

Vesicle fusion is an indispensable cellular process required for eukaryotic cargo delivery. The Sec/Munc18 protein Munc18c is essential for insulin-regulated trafficking of glucose transporter4 (GLUT4) vesicles to the cell surface in muscle and adipose tissue. Previously, our biophysical and structural studies have used Munc18c expressed in SF9 insect cells. However to maximize efficiency, minimize cost and negate any possible effects of post-translational modifications of Munc18c, we investigated the use of *Escherichia coli* as an expression host for Munc18c. We were encouraged by previous reports describing Munc18c production in *E. coli* cultures for use in *in vitro* fusion assay, pulldown assays and immunoprecipitations. Our approach differs from the previously reported method in that it uses a codon-optimized gene, lower temperature expression and autoinduction media. Three N-terminal His-tagged constructs were engineered, two with a tobacco etch virus (TEV) or thrombin protease cleavage site to enable removal of the fusion tag. The optimized protocol generated 1–2 mg of purified Munc18c per L of culture at much reduced cost compared to Munc18c generated using insect cell culture. The purified recombinant Munc18c protein expressed in bacteria was monodisperse, monomeric, and functional. In summary, we developed methods that decrease the cost and time required to generate functional Munc18c compared with previous insect cell protocols, and which generates sufficient purified protein for structural and biophysical studies.

## Introduction

Sec/Munc18 (SM) and SNARE proteins are essential for vesicle exocytosis in eukaryotes [1]–[7]. The assembly of the fusogenic SNARE complex is regulated in part by SM proteins through interaction with their cognate SNARE Syntaxin (Sx), in distinct vesicle transport pathways [3], [8]–[15]. Deletion and over-expression of SM proteins have shown both positive and negative effects in each step of vesicle fusion [3], [16]–[18]. Three of the seven SM isoforms expressed in mammals are involved in exocytosis: Munc18a, Munc18b and Munc18c [19]–[21]. Munc18a is expressed specifically on neuronal cells and is required for neurotransmission. By contrast, Munc18c is ubiquitously expressed [22] and is required for GLUT4 translocation to the cell surface in adipose/muscle tissues in response to insulin signaling, and is also important in endothelial cell activation [23]–[27].

While Munc18 proteins are essential components of vesicle fusion their mode of regulating vesicle fusion is poorly understood [4], [6], [10], [17], [28]. Thus, Munc18:Sx complexes have been reported to inhibit SNARE complex formation [12], [13], [29]–[32] or permit SNARE complex formation [33]–[38]. We are interested in unraveling the role of Munc18c in insulin-stimulated membrane fusion [23], [27], [29], [33], [35], [39]–[43] and have previously used recombinant Munc18c expressed in baculovirus infected insect cells [44] for our structural and biophysical studies. However, this approach is relatively expensive and time-consuming to generate the milligram quantities required on a regular basis, and we cannot rule out the possibility that the produced protein has unintended post-translational modifications that affect its interactions with partner proteins.

Several groups have reported the production of recombinant Munc18c using an *E. coli* expression system to generate recombinant Munc18c protein for their studies (summarized in **Table 1**). For example, Munc18c has been cloned into pQE30 (creating an N-terminal His_6_ fusion protein) and co-expressed with GroEL in M15 *E. coli* cells for *in vitro* pull-downs and liposome fusion assays [29]. Others have used bacterially expressed Munc18c for specific assays (see Table 1 for a summary). However, using the same methods, we were unable to produce sufficient recombinant full-length Munc18c protein for our structural biology and biophysical studies.

**Table 1:** Reported Munc18c expression and purification.

Origin	Residues	Construct	Expression	Purification	Yield	Used for	Reference
Mouse cDNA	FL (1-592)	Munc18c- His_6_	*E.coli* BL21 (DE3)	IMAC (Ni-NTA)	NR^*^	PDA or IP	[43]
Mouse cDNA	FL (1-592)	pET28a-His_6_-Munc18c	*E.coli* cells	IMAC (Ni-NTA)	NR^*^	PDA or IP	[41], [49]
NR^*^	FL (1-592)	pQE30-His_6_-Munc18c	Co-expressed with GroEL in *E.coli* M15 cells, Media NR; IPTG induction at 25°C	IMAC (Ni-NTA)	NR^*^	*In-vitro* fusion assays,	
PDA	[29], [39]						
Rat Munc18c	FL	pGEX-KG-Munc18c	*E.coli* BL21 (DE3) (RIPL) with IPTG (100 nM) induction at 27°C	Protein was purified using glutathione Sepharose beads for GST-moiety	NR^*^	PDA	[40]
Human Munc18c cDNA	FL (1-592)	pQE-9-His_6_-Munc18c	Expressed in *E.coli*	Protein produced in inclusion body was solubilized by 8 M urea and purified on Ni-NTA beads	NR^*^	IP	[26]
Mouse Munc18c	FL (1-592)	pAc-HLT-B-His_6_-Munc18c or pFast-Bac-His_6_-TEV-Munc18c	Insect Sf9 cells	IMAC (TALON) followed by SEC	2–4 mg/L	Purification and Characterization; PDA, SAXS, cross-linking, ITC; *in-vitro* fusion assays	[33], [35], [38], [44]
Mouse (codon optimized synthetic gene)	FL (1-592)	pQE30-His_6_-Munc18c	Co-expressed with GroEL in *E.coli* BL21 cells, auto-induction (ZYP) media at 16°C	IMAC (PrepEase) followed by SEC and IEC	1–2 mg/L	Purification and Characterization; ITC, PDA	This work

NR*- Not reported, **FL**- full-length, **PDA**-pull down assays; **IP-** immunoprecipitation; **ITC**- isothermal titration calorimetry; **SAXS**-small angle X-ray scattering.

Here we report the large-scale production of purified recombinant Munc18c from a codon optimized full-length synthetic mouse gene. We followed the lead of Brandie *et al.*, 2008, by using a pQE30 vector and co-expressing Munc18c with GroEL/GroES chaperones to assist folding. The optimal expression conditions in *E. coli* BL21 cells include the use of auto-induction media and a very low expression temperature (16°C), which delivers 1 mg of recombinant purified Munc18c proteins (with different tags) per L culture. The recombinant protein was folded, stable, monomeric, mono-disperse and functional. By optimizing the expression in *E. coli* we developed a protocol and constructs to generate the amounts of purified Munc18c required for future biophysical and structural studies.

## Materials and Methods

### Munc18c constructs

**Figure 1:**
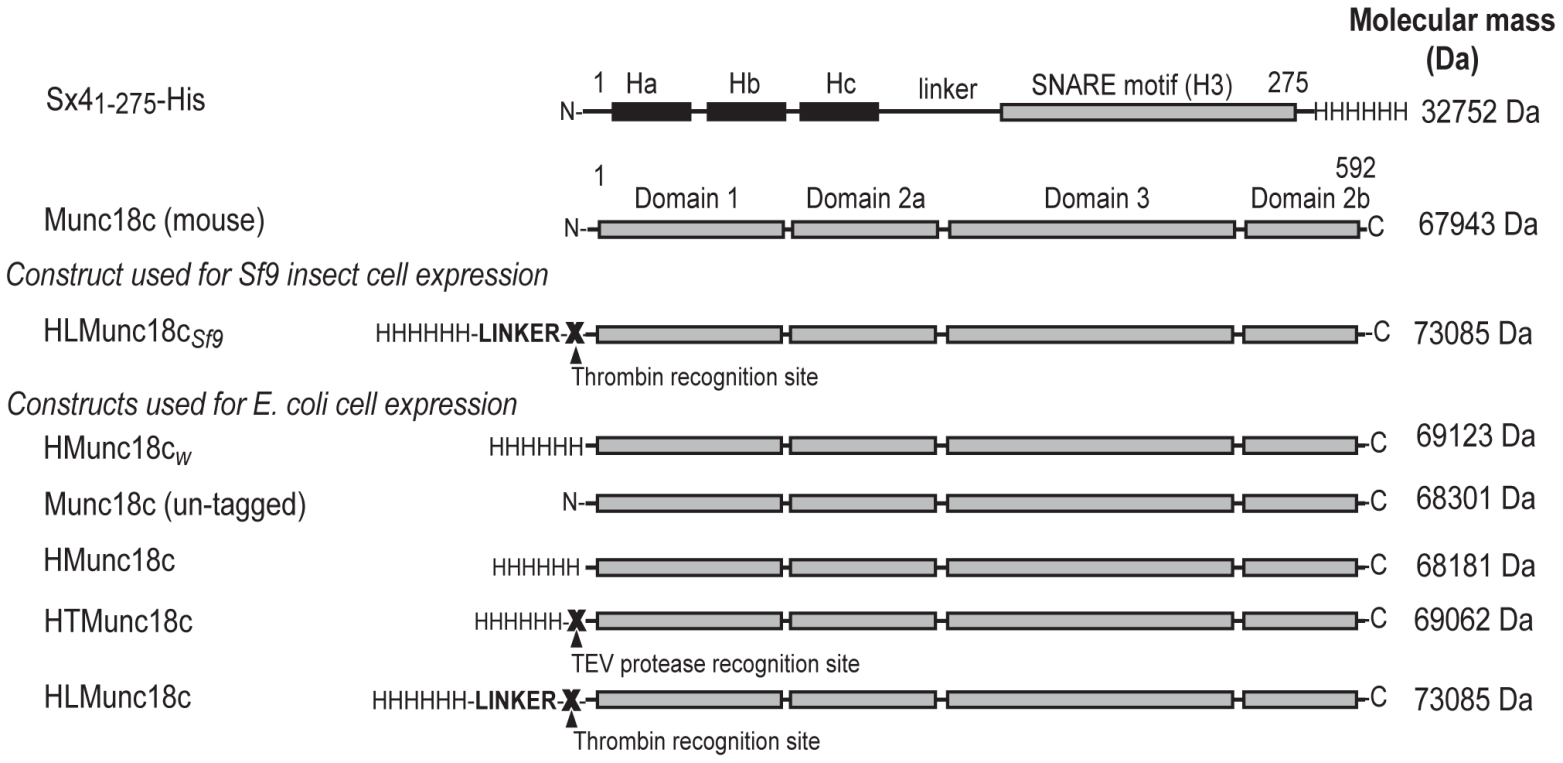
Munc18c and Sx4 constructs. The constructs used in this work or referred to in the text are shown, including the names, fusion tags and molecular mass of each construct. The sequence of the His_6_ linker for HLMunc18c*_Sf9_* and HLMunc18c is given in the text.

**Fig. 1** provides a summary of the constructs used in this work. The wild type Munc18c gene was PCR-amplified from the previously described pAcHLT-B-Munc18c construct [44] and was first sub-cloned into a pGEX vector (GE Healthcare, UK) between *BamHI* and *SmaI* (New England BioLabs, USA) restriction sites using specific forward (5′- CGCGGATCCGGACTGAAGAGCGTC-3′) and reverse primers (5′- GCACCCGGGTTATCACTCATCCTTAAAGG-3′). To generate a construct similar to that described by Brandie et al., (2008), the pGEX-Munc18c and pQE30 plasmids were digested with *BamHI* and *SmaI* (New England Biolabs, USA) and the excised Munc18c gene was inserted into the pQE30 vector, and ligated using T4-ligase (New England Biolabs, USA). This construct is referred to as HMunc18c*_w_* (**Fig. 1**), and was verified by sequencing using T5 forward (*5′-CCCGAAAAGTGCCACCTGATG-3′*) and T5 reverse primers (*5′-GTTCTGAGGTCATTACTGG-3′*). All cloning work involving the pQE30 plasmid was carried out using cell strains carrying a *lacI^q^* mutation (Novablue (Novagen)) or 5-alpha F' *I^q^* (New England BioLabs, USA)).

A synthetic *Mus musculus* Munc18c gene encoding the full-length protein (1-592), optimized to match *E. coli* codon usage patterns, was purchased (GenScript, NJ) (**Fig. S1A**). The synthetic Munc18c gene was supplied in the pUC57 vector, and was excised by digestion with *BamHI* and *SalI* restriction enzymes (New England BioLabs, USA). The pQE30 vector was prepared by digestion with the same restriction enzymes, and the Munc18c gene was ligated into the pQE30 vector with T4 ligase (New England BioLabs, USA). This construct is referred to as HMunc18c (**Fig. 1**), and its sequence was verified as described above.

To permit removal of the His_6_ fusion tag, the coding sequence for a TEV protease cleavage site was inserted between the His_6_ and the Munc18c coding regions. The TEV site was introduced by PCR with 5′ phosphorylated primers *5′-TTCCAATCCATGGCACCGCCGGTTAGC-3′* and *5′-GTACAGGTTCTCGTGATGGTGATGGTGATG-3′* (the coding region for the TEV site is underlined). Linear PCR products were then circularised via blunt end ligation using T4 ligase (New England BioLabs, USA). This construct is referred to as HTMunc18c (His-TEV-Munc18c - **Fig. 1**), and its sequence was verified as described above.

To mimic the construct used for insect cell expression [44], the pQE30-Munc18c plasmid was modified to produce the cleavable fusion sequence MSPIDPMGHHHHHHGRRASVAAGILVPRGSPGLDGIYARGIQASMAAGFG (thrombin recognition site is underlined). To insert this linker in the plasmid, pQE30-Munc18c was digested with *EcoRI and BamHI* to remove the coding region for the original His_6_ tag. The coding sequence for the linker (purchased from Genscript, NJ – See **Fig. S1B**) was also digested with *EcoRI* and *BamHI* and then ligated into the digested pQE30-Munc18c plasmid using T4-ligase (New England BioLabs, USA). This construct is referred to as HLMunc18c (His-Linker-Munc18c - Fig. 1), and its sequence was verified as described above.

An untagged Munc18c construct was generated by PCR amplification, using pQE30-HMunc18c as the template and the primers *5′-CATCACCATCACCATCACGGATCC-3′*, *5′-GCGGGATCCCGATCCTCTCATAGT-3′* (*BamH1* restriction sites underlined). The PCR product was then digested using *BamHI* and then circularised using T4-ligase (NEB, USA). This construct is referred to as Munc18c (un-tagged) (Fig. 1), and its sequence was verified as described above.

### Test Expression in *E. coli*

The plasmid encoding HMunc18c_w_ or HMunc18c was co-transformed with the pREP4-GroESL plasmid [45] into *E. coli* M15 or BL21 for test expression. Transformation mixtures (50 µL) were plated onto Luria Bertani (LB) agar, and selective pressure for clones containing both the pQE30-Munc18c and pREP4 plasmids was applied with ampicillin (100 µg/mL) and kanamycin (50 µg/mL). After 24 h, a single colony was used to inoculate 5 mL LB containing both ampicillin and kanamycin. The culture was incubated at 37°C with shaking overnight. For test expression, 1 L of media (LB, terrific broth (TB) media or ZYP-5052 auto-induction [46] containing ampicillin (100 µg/mL) and kanamycin (50 µg/mL) was inoculated with overnight culture (1 mL). All cell cultures were grown at 37°C to an optical density at 600 nm (OD_600_) of 0.6. LB and TB cultures were then induced with 1 mM IPTG and incubated at 25°C, 20°C or 16°C whilst auto induction cultures were directly incubated at 25°C, 20°C or 16°C. Cell growth in LB and TB media at 16°C was very slow and these were not continued further. After incubation for 20 to 22 h, cells were harvested by centrifugation (JLA 8.1 rotor, AVANTI centrifuge (Beckman Coulter, USA), 5000 g, 15 min at 4°C). Pelleted cells (1 L cell pellets) were resuspended in Tris lysis buffer (25 mM Tris-HCl pH 7.5; 300 mM NaCl, 10% (w/v) glycerol, 1% (w/v) Triton X-100, 5 mM imidazole, 2 mM 2-mercaptoethanol (β-ME), 10 mM MgCl_2_) at a ratio of 10 mL per gram of wet pellet. To this mixture, 12,500–14,000 units DNase (Roche) and 100 µL of Bacterial Protease Inhibitor (BioPioneer, Inc., USA) was added, and the cells then lysed by sonication (10 Hz pulses for 30 sec at 30% amplification on an Ultrasonic homogenizer (BioLogics Inc. Australia)). Cell debris was removed by centrifugation (JA25.5 rotor, AVANTI centrifuge (Beckman Coulter, USA), 18,500 g, 40 min, 4°C) and the soluble fraction was incubated with 0.5 mL of Co^2+^-affinity beads (TALON beads, Takara Bio Inc, Japan) pre-equilibrated in Tris wash buffer (25 mM Tris-HCl pH 7.5; 300 mM NaCl, 10% (w/v) glycerol, 20 mM imidazole, 2 mM β-ME) per 100 mL lysate for 2 h at 4°C to bind the His_6_-tagged Munc18c. After 2 h, the beads were washed in five column volumes of Tris wash buffer and protein was eluted in 1 mL aliquots using Tris elution buffer (Tris wash buffer plus 300 mM imidazole). Samples were analysed by SDS-PAGE gels.

### Large-Scale Expression of Munc18c in *E. coli*

For large-scale protein production, 2–6 L cultures were used. The plasmid vector encoding the codon-optimized Munc18c gene (HMunc18c, HTMunc18c, HLMunc18c or untagged Munc18c) was freshly co-transformed into *E. coli* BL21 with the pREP4 plasmid encoding the GroEL/ES chaperones, and grown in culture as described above using autoinduction and moving cultures from a 37°C incubator to a 16°C incubator after OD_600_ 0.5–0.6 was reached. Cell pellets were harvested by centrifugation as described above, weighed, frozen in liquid nitrogen and stored at −80°C until used for purification. Some variation in expression levels was observed using different colonies, so glycerol stocks were made from the best-expressing colonies for inoculating large-scale expression cultures and these routinely gave the reported expression yields.

### Large Scale Purification of HMunc18c or HLMunc18c

Cell pellets (∼15–20 g/L) were thawed on ice for 30 min and then homogenised into a 1∶10 ratio (wet cell pellet mass: lysis buffer) in Munc18c lysis buffer (25 mM Tris-HCl pH 7.5, 300 mM NaCl, 10% (v/v) glycerol, 10 mM imidazole, 2 mM βME, 1% (v/v) Triton X-100, 0.5 mM EDTA) with 100 µL of Bacterial Protease Inhibitor (BioPioneer, Inc., USA), on ice. The cells were homogenised by multiple passes through a 30 mL disposable syringe and lysed by addition of lysozyme (Astral Scientific, Australia) to a final concentration of 400 µg/mL and incubated at 4°C for 1 h. To reduce viscosity, ∼13,000 U of DNase (Roche, Australia) and 1 mM MgCl_2_ and 1 mM CaCl_2_ was added and the solution was incubated for a further 1 h at 4°C with mixing. The cell lysate was centrifuged to remove the cell debris (JLA 16.25 rotor, AVANTI centrifuge (Beckman Coulter, USA), 13,500*g*, 30 min, 4°C). The supernatant was mixed with Ni-chelated PrepEase™ resin (USB Corporation, USA) pre-equilibrated with wash buffer (25 mM Tris-HCl pH 7.5, 300 mM NaCl, 10% (v/v) glycerol, 10 mM imidazole, 2 mM β-ME) and incubated for 2 h at 4°C with gentle mixing. After incubation, the beads were loaded into a gravity column (Maxi Column, G Bioscience, USA) and washed with 50 mL of the Tris-HCl wash buffer, followed by 50 mL of Tris-HCl wash buffer containing 25 mM imidazole. After washing, the protein was eluted in 1 mL fractions in elution buffer (Tris-HCl buffer with 300 mM imidazole). Eluted protein was analysed on SDS-PAGE (described below) and fractions containing Munc18c were pooled, concentrated to a total volume of 6 mL (10 kDa MWCO centrifugal concentrator (Amicon Merck, Germany) and injected onto a pre-equilibrated Superdex 200 16/60 (S200) size exclusion chromatography (SEC) column on an ÄKTA FLPC system (GE Healthcare, UK) in SEC Buffer (25 mM HEPES pH 8.0, 200 mM NaCl, 2 mM β-ME, 10% (v/v) glycerol). Fractions containing Munc18c were pooled, concentrated (as before) and injected onto a MonoS cation exchange 5/50 column (pre-equilibrated with buffer A) and purified using a salt gradient from 0%–80% Buffer B (25 mM HEPES pH 8.0, 1000 mM NaCl, 2 mM β-ME, 10% (v/v) glycerol) over 50 Column Volumes at a flow rate of 1 mL per min. Munc18c eluted at between 15–20% of buffer B. Peak fractions were assessed by SDS-PAGE, and highly homogeneous fractions were pooled and concentrated to the desired protein concentration and stored at −80°C.

### Large Scale Purification of detagged HTMunc18c

To produce Munc18c lacking a His_6_ fusion tag, HTMunc18c was expressed and purified to the point of elution from the PrepEase™ resin, as described above for HMunc18c and HLMunc18c. The eluted HTMunc18c was mixed with TEV protease [47] (0.1 mg protease per 10 mg of protein) was placed in 6–8 kDa MWCO dialysis tubing (Spectrum Lab, Inc. USA) and dialysed overnight against SEC buffer at 4°C incubated in 6–8 kDa MWCO dialysis tubing (Spectrum Lab, Inc. USA). The resulting mixture (HTMunc18c, de-tagged Munc18c, His_6_-TEV, His_6_) was collected from the tubing and incubated with equilibrated PrepEase™ beads in HEPES wash buffer (25 mM HEPES pH 8.0, 300 mM NaCl, 10% (v/v) glycerol, 2 mM βME, 10 mM imidazole) at 4°C for 30 min to separate de-tagged Munc18c (in solution) from His_6_-TEV, His_6_ and any remaining HTMunc18c (which should all be bound to the resin). The resin was placed in a gravity column, and the flowthrough containing the de-tagged Munc18c was collected. Purity of the de-tagged Munc18c was assessed by SDS-PAGE. Fractions containing the de-tagged protein were pooled and injected onto the pre-equilibrated Superdex-200 16/60 column in SEC buffer on an ÄKTA FPLC™ system (GE Healthcare, UK). Peak fractions were collected, analysed on SDS-PAGE before pooling and concentrating fractions containing Munc18c and storing at −80°C as described above.

### Purification of Syntaxin4 (Sx4_1-275_-His)

C-terminally His_6_-tagged Sx4 C141S (residues 1-275) (Sx4_1-275_-His) was produced as described previously [33]–[35], [44].

### Interaction of Munc18c with Sx4_1-275_-His and with assembled SNARE complex

To determine whether the Munc18c recombinant protein expressed in *E. coli* was functional, an *in vitro* binding assay with the cognate *t*-SNARE binding partner Sx4 was performed. In this assay, purified Sx4_1-275_-His protein (30 µg) was incubated with 50 µL TALON resin pre-equilibrated with 100 µL of binding buffer (25 mM HEPES pH 8, 300 mM NaCl, 2 mM βME, 10% (v/v) glycerol, 10 mM imidazole pH 7.5, 0.05% (v/v) Triton X-100) for 2 h at 4°C. To remove unbound protein, the beads were washed three times with 500 µL wash buffer (binding buffer with 20 mM imidazole). The immobilised Sx4_1-275_-His was then mixed with 50 µg purified detagged Munc18c in binding buffer and incubated at 4°C with slow mixing for 30 min, 60 min, 120 min and overnight. After incubation, the beads were washed three times with 200 µL of wash buffer. For the negative control, purified de-tagged Munc18c protein (50 µg) alone was incubated with beads in the same manner overnight at 4°C. After washing, beads were mixed with SDS-loading buffer (50 µL) and samples were denatured at 95°C for 5–10 min prior to loading onto a 4–12% Nu-PAGE Bis-Tris SDS-PAGE gel.

The binding of Sx4_1-275_ to recombinant mouse Munc18c expressed in *E. coli* and recombinant mouse Munc18c expressed in insect cells (HLMunc18c_Sf9_) [44] was also determined by monitoring the intrinsic tryptophan fluorescence (excitation 280 nm and emission 310–400 nm) using a Synergy H1-BioTech plate reader. As Sx4_1-275_ does not contain any tryptophan residues, any change in overall fluorescence is likely due to conformational changes in Munc18c upon binding Sx4. All proteins were buffer exchanged into 25 mM HEPES pH 8.0, 300 mM NaCl to remove β-ME using G-25 NAP columns (GE Healthcare, UK). The fluorescence was measured for buffer alone (25 mM HEPES pH 8.0, 300 mM NaCl) and then for each of the purified proteins (HMunc18c, HLMunc18c_Sf9_, or Sx4_1-275_-His) at a concentration of 500 nM. The tryptophan fluorescence spectrum was then measured for HMunc18c/Sx4_1-275_-His and HLMunc18c_Sf9_/Sx4_1-275_-His (with each protein at 500 nM, in a 100 µL reaction volume). The change in the intrinsic fluorescence upon binding of Munc18c to Sx4 was monitored for each sample in triplicate.

The ability of purified Munc18c to interact with assembled SNARE complex was assessed using the protocol described previously [35]. Briefly, purified SNARE proteins were purified, mixed in a 1∶1∶1 molar ratio and incubated overnight at 4°C. TALON beads were added to pull down the SNARE complex through interaction with the His_6_ tag of Sx4_1-275_-His. The beads were washed three times in wash buffer 1 containing 0.1% (v/v) Triton X-100 and then incubated for 2 h at 4°C with de-tagged Munc18c. Beads were washed a further three times in the same wash buffer, mixed with loading dye, boiled for 10 min and bound proteins analysed by reducing SDS-PAGE.

### Isothermal Titration Calorimetry (ITC)

ITC experiments were carried out at 298 K using an iTC200 (Microcal) instrument to assess the thermodynamics of Munc18c and Sx4 interaction. The proteins (HMunc18c or Sx4_1-275_-His) were purified as described above and buffer exchanged into ITC buffer (25 mM HEPES pH 8, 200 mM NaCl, 10% (v/v) glycerol and 2 mM β-ME) by gel filtration prior to measurements. Sx4_1-275_-His at a concentration of 200–220 µM was titrated into 20–30 µM of HMunc18c in the cell. Injection volumes of 2.8 µL were used for all titrations. The heat released was measured and integrated using the Microcal Origin 7.0 program using a single site binding model to calculate the equilibrium association constant *K*_a_ ( = 1/*K*_d_), enthalpy of binding (Δ*H*) and the stoichiometry (*n*). The Gibbs free energy (Δ*G*) was calculated using the equation Δ*G* = −RT*In*(*K*_a_); binding entropy (Δ*S*) was calculated by Δ*G* = Δ*H*−TΔ*S*. Four replicates were used to generate mean and standard error of the mean (SEM) values.

### Purification of the Munc18c/Sx4_1-275_-His complex

Recombinant un-tagged Munc18c and Sx4_1-275_-His proteins were expressed in bacteria as described above. The lysates were mixed in a 3∶1 (Munc18c∶Sx4) volume ratio to give an estimated molar excess of Munc18c (assuming Munc18c expression ∼3 mg/L; Sx4_1-275_-His expression ∼3–4 mg/L) and the mixture was incubated on ice for 1–2 h. The mixed lysates were cleared by centrifugation using a JA 25.5 rotor in an AVANTI centrifuge (Beckman Coulter, USA) at 18,500*g*, for 40 min, 4°C). The mixed cleared lysates (200 mL) were then added to TALON affinity beads (1 mL) equilibrated in binding buffer (25 mM Tris-HCl pH 7.5, 300 mM NaCl, 10% (v/v) glycerol, 2 mM β-ME, 0.1% (v/v) Triton X-100), to which 100 µL of Bacterial Protease Inhibitor (BioPioneer, Inc., USA)and ∼13,000 U of DNase (Roche, Australia) was added, and this mixture was then incubated at 4°C for 2 h with mixing. The beads were then washed with 150 mL wash buffer 1 (25 mM Tris-HCl pH 7.5, 300 mM NaCl, 10% (v/v) glycerol, 2 mM β ME, 0.01% (v/v) Triton X-100, 10 mM imidazole) followed by 150 mL wash with wash buffer 2 (wash buffer 1 without Triton X-100). The protein bound to beads was eluted in 1 mL fractions with elution buffer (25 mM Tris-HCl pH 7.5, 300 mM NaCl, 10% (v/v) glycerol, 2 mM β-ME, 300 mM imidazole). The eluted protein was then concentrated using a 10 kDa MWCO concentrator (Amicon, Merck, Germany), injected (5 mL) and purified on a Superdex200 16/60 column on ÄKTA FPLC™ system pre-equilibrated in SEC buffer (25 mM Tris-HCl pH 7.5, 300 mM NaCl, 10% (v/v) glycerol) and analysed by SDS-PAGE.

### Analytical Size Exclusion Chromatography (SEC)

To assess the homogeneity and stoichiometry of Munc18c/Sx4 complexes an analytical grade Superdex200 10/300 GL column (GE Healthcare, UK) was pre-calibrated with the following molecular mass standards: beta-amylase (200 kDa), alcohol dehydrogenase (150 kDa), albumin (66 kDa); carbonic anhydrase (29 kDa) and cytochrome C (12.4 kDa) (Sigma Aldrich, USA). Proteins alone (HMunc18c, Sx4_1-275_-His) or in complex (un-tagged Munc18c: Sx4_1-275_-His complex) were buffer exchanged into 25 mM Tris-HCl pH 7.5, 300 mM NaCl, 10% (v/v) glycerol and concentrated to 3–5 mg/mL using 3 kDa (for Sx4), 10 kDa (for Munc18c) and 30 kDa for complex) MWCO concentrator (Amicon, Merck, Germany). For analysis, 500 µL of each sample was injected onto the column (pre-equilibrated in the same buffer) and analysed at a flow rate of 0.5 mL/min with column pressure 1.5 MPa. Peak fractions with the correct apparent molecular mass (based on molecular mass standard calibration) were collected, analysed by SDS-PAGE and stored at 4°C for further analysis.

Multi-angle light scattering with SEC (SEC-MALS) was performed at room temperature. A sample (500 µL) of purified HMunc18c at a concentration of 2.5 mg/mL was injected onto an S200 10/300 GL analytical column attached to a mini Dawn laser light scattering photometer and Optilab DSP interferometric refractometer (Wyatt Technology, USA). The column was pre-equilibrated with buffer containing 25 mM Tris-HCl pH 7.5, 300 mM NaCl, 2 mM βME, 10% glycerol. Bovine serum albumin (BSA, Sigma) was used as an isotropic scatterer for detector normalisation. Mass estimation was determined by Debye fitting.

### SDS-PAGE Analysis

SDS-PAGE (Laemmli, 1970) was used for sample analysis. Samples (20 µL) collected after each purification step were dissolved in BioRad Laemmli Sample Buffer with addition of 30 mM DTT. Samples were then denatured by heating at 95°C for 10 min and loaded onto 4–12% NuPAGE Bis-Tris gels (Life Technologies, USA). Gels were run in MES buffer at constant 200 mV for 35–40 min. Coomassie Brilliant R250 Blue stain (Sigma Aldrich, USA) was used to visualize protein bands on the gels. Low molecular weight marker (GE Healthcare, UK, Catalog No.: 45-000-072) was used for all SDS-PAGE gels.

### Mass Spectroscopy

Mass spectrometry MALDI TOF/TOF was used to identify protein contaminants following in-gel tryptic digestion of SDS-PAGE bands corresponding to masses of ∼30 kDa and ∼21 kDa (as compared with standard SDS-PAGE molecular marker). The protein bands were cut from the gel and each band was dehydrated in 1.0 mL of 100% methanol for 5 min at room temperature, rehydrated in 1.0 mL of 30% methanol for 5 min and destained with 3 washes in 1.0 mL 100 mM NH_4_HCO_3_ containing 30% acetonitrile. The gel pieces were cut into ∼1 mm cubes and washed 3 times in 1.0 mL sterile water then dried in an Alpha-RVC vacuum apparatus for 30 min. Sufficient trypsin (5–10 ng/µL) (Promega, USA) in 50 mM NH_4_HCO_3_ was added to cover the dried gel pieces. Trypsin digestion was carried out by incubating the gel pieces at 37°C overnight. The samples were centrifuged (microfuge (ThermoFisher Scientific, USA), 14,000 g, 2 min) and the supernatant collected, the tryptic peptides were then serially extracted from the gel pieces with 50% acetonitrile containing 0.1% formic acid. The extracted peptide fractions were combined and dried using an Alpha-RVC vacuum concentrator (ThermoFisher Scientific, USA). Samples were reconstituted in 50% acetonitrile containing 0.1% formic acid and mixed 1∶1 with matrix (5 mg/mL α-cyano-hydroxy-cinnamic acid, in 50% acetonitrile, 0.1% formic acid and 2 mM ammonium phosphate) on a MALDI target plate. MALDI-TOF/TOF mass spectrometry (4700 Proteomics Analyser) was performed to gather mass and sequence information for the peptides.

## Results

### Optimization of recombinant Munc18c production from bacterial culture

**Figure 2:**
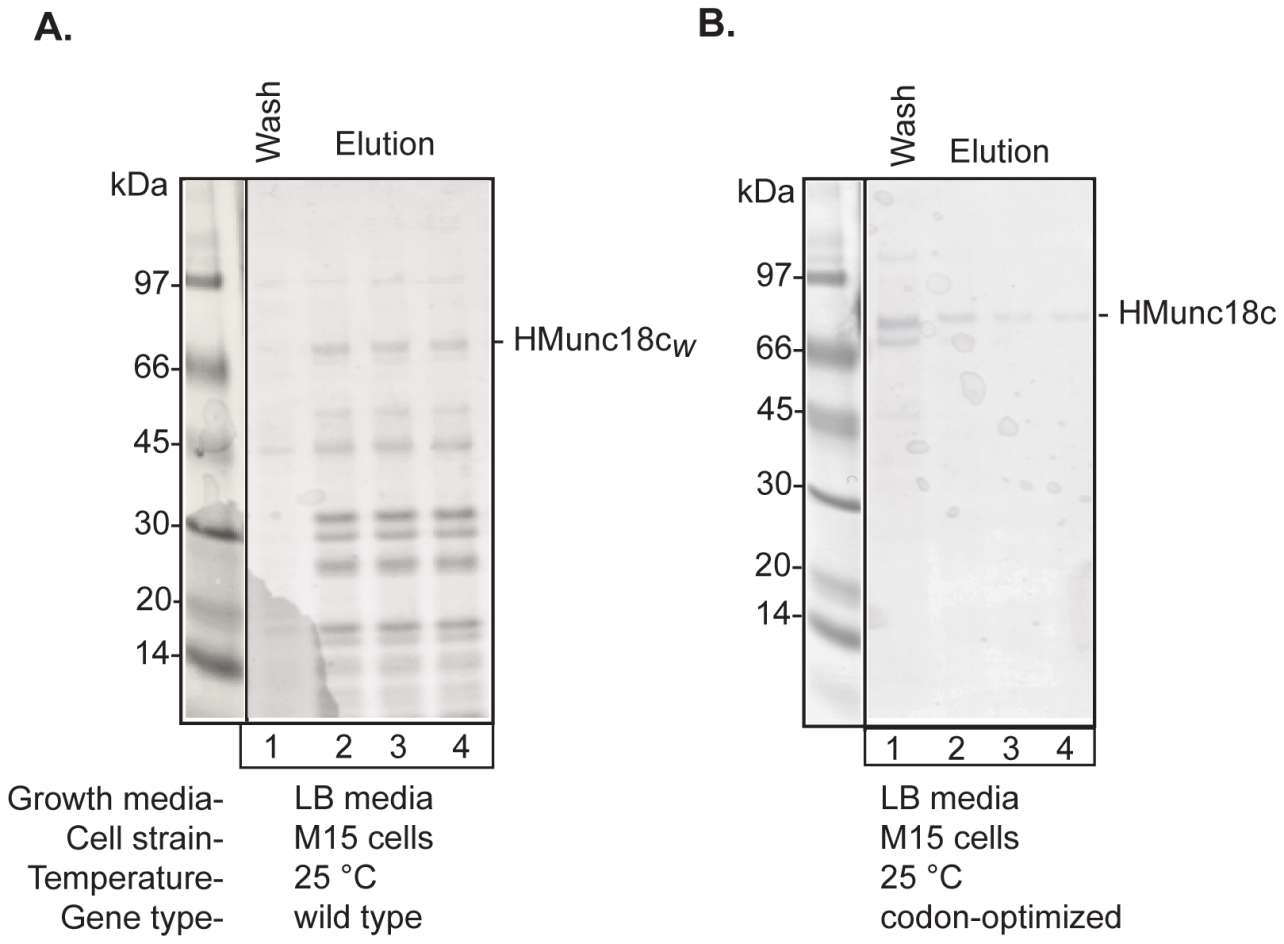
Purification of full-length Munc18c from test expressions in *E. coli* cultures. Coomassie stained SDS-PAGE showing purification of Munc18c from test expressions. **A.** HMunc18c*_w_*, using conditions similar to those described in Brandie et al., (2008). Wash and elution fractions from beads are shown. **B.** HMunc18c, using the same conditions.

The production of recombinant Munc18c from a bacterial expression host has been reported previously (Table 1). We were interested in using bacterially expressed Munc18c for structural and biophysical experiments that require mg quantities of highly purified protein. However, to the best of our knowledge, the yield of protein using these bacterial expression systems was not reported, so it was not clear whether these methods would suffice for our requirements. Following the lead of Brandie et al., (2008) we tested Munc18c expression using the same approach, by co-expressing mouse HMunc18c*_w_* (native DNA sequence) with GroEL/GroES in M15 *E. coli* cells at 25°C. The media was used by Brandie et al (2008) to express Munc18c was not explicitly stated; we used LB media. Under these conditions, we observed that a band consistent with HMunc18c eluted from affinity beads, though with significant levels of impurities (**Fig. 2A**). We therefore explored several modifications to the procedure to optimize yield of HMunc18c.

**Figure 3:**
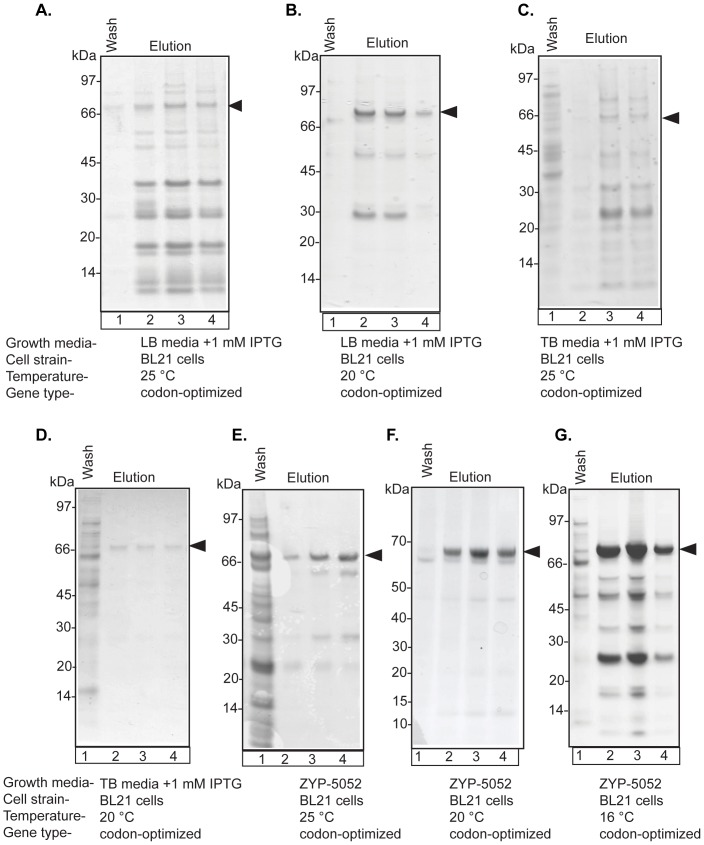
Expression optimization of codon-optimized full-length HMunc18c in *E. coli* cultures. Different media, cell expression strains and expression temperatures (as indicated) were used to optimise yield of HMunc18c using 1 L cultures. All cultures were grown at 37°C until OD_600_ reached 0.5–0.6 and then either induced with 1 mM IPTG (LB and TB) and/or temperature lowered. **A.** BL21 strain, LB Media, 25°C. **B.** BL21 strain, LB media, 20°C. **C.** BL21 strain, TB media, 25°C. **D.** BL21 strain, TB media, 20°C. **E.** BL21 strain, ZYP-5052, 25°C. **F.** BL21 strain, ZYP-5052, 20°C. **G.** BL21 strain, ZYP-5052, 16°C. The black arrow indicates the expected band for HMunc18c.

First, we used a Munc18c gene in which the codon usage patterns were altered to match that of *E. coli*. Under the same conditions, (*i.e.* in the same plasmid as HMunc18c, in M15 cells at 25°C), a band corresponding to Munc18c was barely discernible on a gel, suggesting very low-level expression, albeit with few if any contaminants (**Fig. 2B**). Expression of the codon-optimized gene was then assessed in LB and TB media with IPTG induction in *E. coli* strain, BL21, at 25 or 20°C (**Fig. 3A–D**). Under these conditions, the BL21 strain using LB media was clearly superior to the M15 cells, showing a much higher level of expression with far fewer contaminants (**Fig. 3A and 3B**). Using the codon-optimized gene in BL21 cells, with ZYP-5052 auto-induction media [46], LB media or TB media at different temperatures, expression at 20°C gave clear evidence of a single purified band on SDS-PAGE (**Fig. 3F**), though levels were inconsistent from batch to batch (not shown). However, by moving the culture to 16°C at OD_600_ 0.6, the yield was increased further using ZYP-5052 media (to between ∼1–2 mg purified protein from 1 L culture, for different constructs), though with much higher levels of contaminants (**Fig. 3G**). For the remaining analyses, we chose to use ZYP-5052 auto-induction media in combination with expression at 16°C after cultures reached OD_600_ 0.6.

Next, protein harvesting and purification were optimised. Initially, cell lysis was performed using sonication but this sometimes resulted in lower molecular weight bands on gels after elution from affinity beads (**Fig. S2A**). To overcome this, a gentler method was developed whereby cells were homogenised in cell lysis buffer at room temperature for 30 min followed by 1 h incubation with lysozyme at 4°C with mixing. This reduced the level of contaminants for HMunc18c (**Fig. S2B**) and all subsequent purifications were treated in this way.

**Figure 4:**
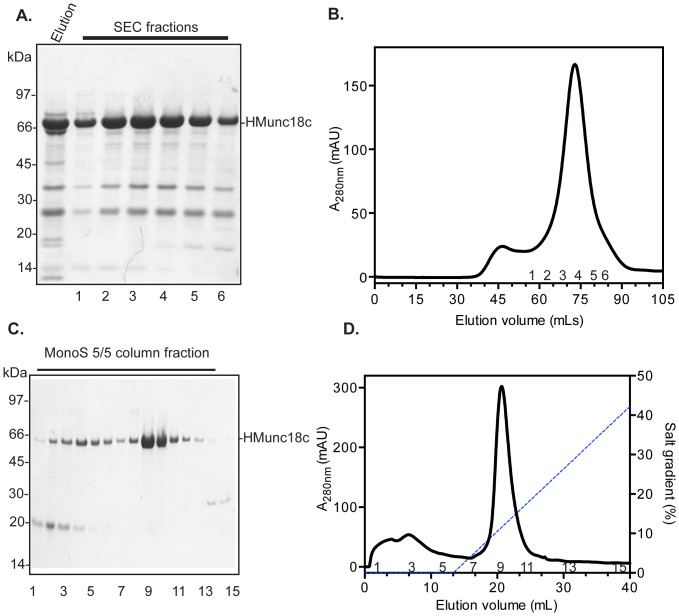
Purification of recombinant HMunc18c expressed in *E. coli* cultures. **A.** SDS-PAGE analysis of HMunc18c purification steps. Elution fraction from IMAC, labelled, was injected onto SEC and eluted as shown in **Lanes 1–6** (which correspond to the labelled fractions in panel B). **B.** Elution profile of HMunc18c from SEC. Peak fractions were pooled and injected onto a MonoS column. **C.** SDS-PAGE of fractions from the MonoS purification step, showing separation of the protein from lower molecular weight contaminants. **D.** Elution profile of HMunc18c from MonoS.

SDS-PAGE of HMunc18c from SEC peak fractions generally revealed lower molecular weight contaminants (**Fig. 4A and 4B**). MALDI TOF/TOF mass spectrometric analysis of the tryptic peptides arising from these bands allowed the identification of the contaminants as bacterial SlyD (∼21 kDa, a protein rich in histidine), and bacterial 50S ribosomal protein (∼30 kDa) (Mascot Peptide Mass Fingerprint as available at www.matrixscience.com). The pI of Munc18c (8.3) was sufficiently different to those of the contaminants (pI 4.9 for SlyD and pI 10.9 for 50S) that it was thought these could be separated by ion exchange chromatography (IEC). Indeed, an IEC step following SEC was able to separate the contaminants (**Fig. 4C and D**), and yielded 1–2 mg purified HMunc18c per L of bacterial cell culture.

Similar optimised expression and purification methods were applied to HTMunc18c and HLMunc18c (**Fig. S3 and S4**). However, IEC was not used for HTMunc18c. Instead the His tag was removed by TEV-protease cleavage and the cleaved product separated from the cleaved His tags and histidine rich contaminant proteins using a reverse IMAC step. The final yield of purified de-tagged HTMunc18c (TEV protease treated), and of purified HLMunc18c (∼1 mg per L, for 1–2 L cultures) was a little lower than for HMunc18c.

### Recombinant HMunc18c from bacterial cultures is monomeric and functional

**Figure 5:**
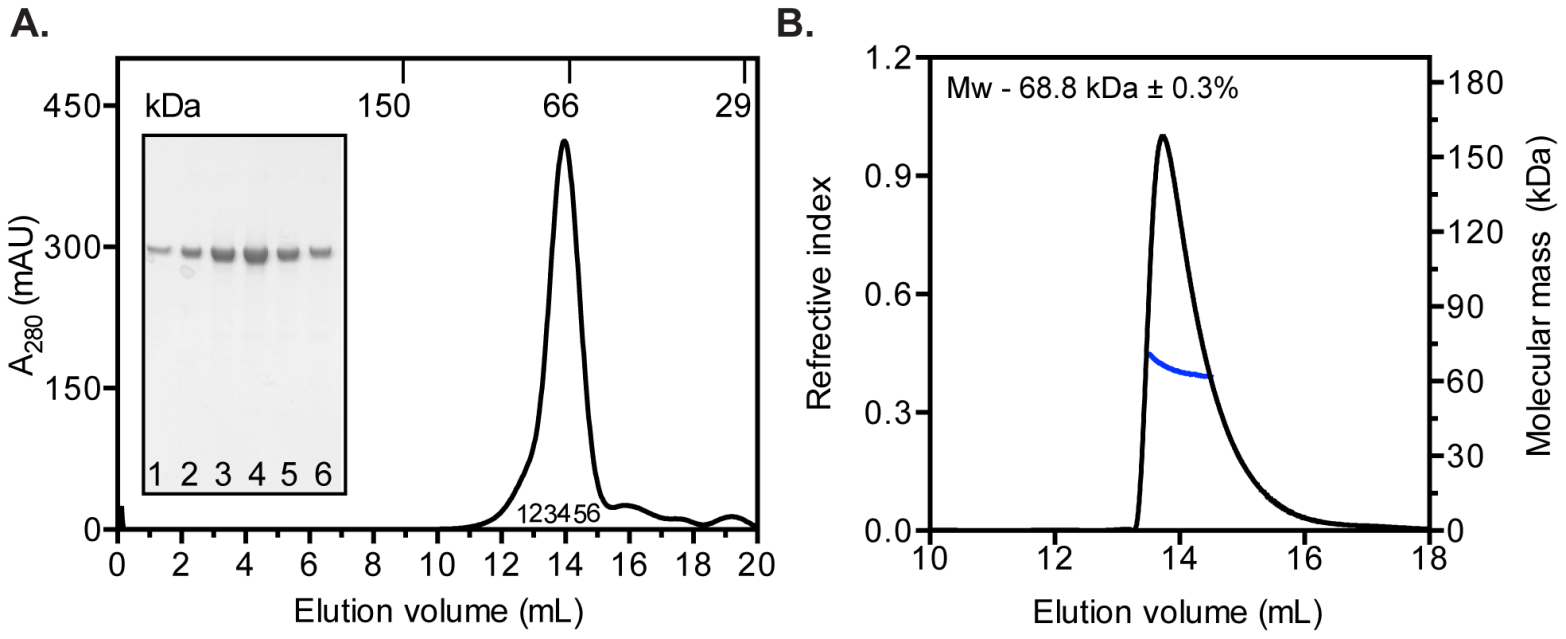
Purified HMunc18c is monomeric in solution. **A**. Elution profile of purified HMunc18c on a calibrated analytical size exclusion chromatography column (S200 10/300 GL). HMunc18c eluted at a volume consistent with a ∼70 kDa protein. Peak fractions were analysed on 4–12% gradient SDS-PAGE (inset). **B**. Elution profile of HMunc18c examined by SEC-MALS. The horizontal blue line corresponds to the SEC-MALS calculated mass (right axis) plotted with the refractive index indicating the peak (left axis) of the protein in the sample (68,200 Da ±0.5%).

Purified HMunc18c was analysed on an analytical Superdex 200 column (GL 10/300) to assess its homogeneity in solution. HMunc18c eluted as a major peak (**Fig. 5A**) at a volume consistent with a 68 kDa protein. SEC-MALS analysis of the purified bacterially expressed HMunc18c gave an estimated mass of 68.8 kDa (±0.3%) confirming that the protein was monomeric (**Figure 5B**) (theoretical mass for monomer, 68,181 Da). Moreover, purified HLMunc18c expressed using either bacterial or baculovirus expression systems had equivalent traces on SEC chromatograms (**Fig. S5**).

**Figure 6:**
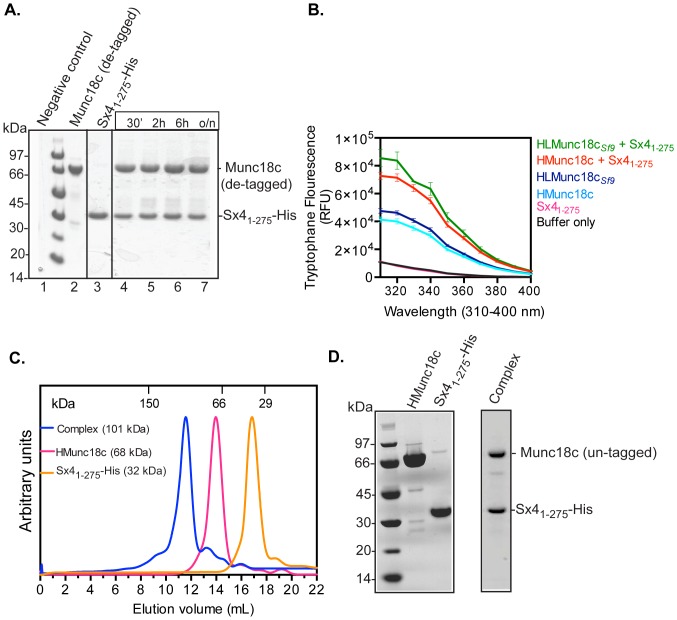
Bacterially expressed Munc18c interacts with Sx4_1-275_-His. **A.** Coomassie Blue stained SDS-PAGE gel showing that bacterially expressed Munc18c (de-tagged) is pulled down by Sx4_1-275_-His immobilised on resin (Lane 4–7). The negative control (Lane 1), shows that Munc18c (de-tagged) is not pulled down by the resin alone. Inputs for the experiment are in Lanes labelled 2 and 3. **B.** The change in intrinsic fluorescence upon mixing Munc18c (expressed in bacteria or insect cells) and Sx4_1-275_-His. A baseline corrected tryptophan fluorescence emission spectra of buffer only, Sx4-_1-275_-His, HMunc18c, HLMunc18c_Sf9_, HMunc18c/Sx4-_1-275_-His, and HLMunc18c*_Sf9_*/Sx4-_1-275_-His complex. **C.** Elution profiles from analytical SEC of Sx4_1-275_-His (dotted line), HMunc18c (dashed line) and the Munc18c:Sx4_1-275_-His complex (solid line) co-purified after mixing lysates of Munc18c (untagged) and Sx4_1-275_-His. The data were normalised to the same peak height. The Munc18c:Sx4_1-275_-His complex eluted at the expected volume for a 1∶1 heterodimer (molecular mass ∼100 kDa). **D.** SDS-PAGE gel showing the input samples of HMunc18c and Sx4_1-275_-His used in panel C, and the eluted complex from panel C confirming the presence of two components.

To test whether the recombinant HMunc18c expressed in *E. coli* cultures was functional, its ability to interact with cognate SNARE partner Sx4, was tested. In an *in vitro* pull down assay, Sx4_1-275_-His was C-terminally immobilized onto affinity beads and incubated with Munc18c (de-tagged HTMunc18c). The results showed that Munc18c expressed in *E.coli* BL21 cells was pulled down by Sx4_1-275_-His on beads within the first 30 min of incubation (**Fig. 6A**).

The Munc18c/Sx4 interaction was further evaluated by intrinsic tryptophan fluorescence. Sx4 does not contain any tryptophan residues, whereas Munc18c has six tryptophan residues. Hence, fluorescence changes upon mixing the two would be indicative of interactions and conformational changes in Munc18c. Fluorescence was measured after mixing HMunc18c and Sx4_1-275_-His (500 nM). The spectra of HMunc18c only and Munc18c:Sx4_1-275_-His complex using Munc18c expressed either in *E.coli* (HMunc18c) or in insect Sf9 cells (HLMunc18c_Sf9_) reveal similar changes (**Fig. 6B**) indicating that bacterially expressed mouse Munc18c behaves similarly to insect-cell expressed mouse Munc18c.

To further explore the interaction, we measured the thermodynamic parameters for the interaction between Munc18c and Sx4_1-275_ using ITC. The dissociation constant for the interaction (*K*_d_) was 104±43 nM (Table 2) (**Fig. S6**), which compares closely to the previously reported affinity using insect cell expressed Munc18c [33]. Overall, three different approaches (pulldown, fluorescence, ITC) show that Munc18c binds strongly to Sx4_1-275_-His independent of the host expression system.

**Table 2:** Thermodynamic parameters of Munc18c/Sx4 interactions (values are shown as a mean from four sets of different experiment ± SEM).

Munc18c/Sx4 thermodynamic parameters							
In cell	Titrant	Δ*H* (kcal/mol)	TΔS (kcal/mol)	Δ*G* (kcal/mol)	K_d_ (nM)	N	Reference
HMunc18c	Sx4_1-275_-His (32 kDa)	−11.0±0.4	5.6±1.4	−9.2±0.5	104±43	0.97	This work
HLMunc18c_Sf9_		−7.70±0.1	−1.9±0.1	−9.5±0.1	95±15	0.98	[33]

The recombinant Munc18c proteins purified from bacterial expression cultures (HLMunc18c and HMunc18c) can be crystallized in the presence of the Sx4 N-peptide (not shown), under conditions used to crystallize Munc18c derived from baculovirus expression [45], [48]. Finally, we showed using a pulldown assay that Munc18c (de-tagged) produced from bacterial expression interacts with pre-assembled SNARE complex (**Fig. S7**), using the same approach described previously for Munc18c produced using baculovirus expression [35].

### Purification of the Munc18c:Sx4 complex

An important goal of current Munc18c research is to obtain a structure of the Munc18c complex with Sx4. This requires the regular production of milligram amounts of the purified complex. We were able to generate the Munc18c:Sx4 complex, by following the same protocols used to produce a Munc18c:Sx4 complex from insect cell expressed Munc18c [44]. The Munc18c: Sx4_1-275_-His complex was formed by mixing the lysates of *E. coli* expressed mouse Munc18c (un-tagged) and Sx4_1-275_-His. The mixed lysates were clarified and then incubated with TALON beads, to pull down Sx4_1-275_-His and bound protein. Eluted fractions from TALON beads were pooled and analysed by SEC. A major peak eluted at a volume consistent with a 100 kDa protein (the mass of the Munc18c: Sx4_1-275_-His complex) (**Fig. 6C**). Peak fractions analysed by SDS-PAGE revealed two bands with relative intensities suggesting the formation of a 1∶1 stoichiometric heterodimer complex between Munc18c and Sx4_1-275_-His (**Fig. 6C**). This result indicates that both proteins Munc18c and Sx4_1-275_-His (expressed in bacteria) are correctly folded and functionally competent to form a stable complex.

## Discussion

The interaction between Munc18 and Sx proteins is a major focus for understanding the molecular basis of vesicle fusion. Research relating to the molecular mechanism and regulation of this key complex requires the ability to produce milligram quantities of the purified, soluble and folded target proteins using rapid, reproducible and cost-effective methods. Structural studies of Munc18c have used recombinant protein expressed in baculovirus-infected insect cells [33], [34], [38], [44] whereas several biochemical studies have used recombinant Munc18c expressed from *E. coli* cultures [29], [39]–[42], [49]. Here, we attempted to optimize the expression of Munc18c in *E. coli* to enable sufficient yields for structural and biophysical studies.

We succeeded in this goal by making use of the previously reported co-expression of Munc18c with GroEL [29] combined with the following modifications: use of codon-optimized gene, BL21 *E. coli* cells, auto-induction media and expression at low temperature. Also, by replacing the sonication step with a gentler lysozyme treatment, we were able to minimize the level of lower molecular weight contaminants of Munc18c at the first stage of purification. The identification of the bacterial contaminants enabled us to add an IEC step to the purification and remove the contaminants by taking advantage of their pI values relative to Munc18c. We were able to generate mg quantities of purified Munc18c sufficient for structural and biophysical studies.

Most importantly, the purified Munc18c was monomeric, mono-disperse and functional. We were able to show that Munc18c binds Sx4_1-275_-His robustly using several different approaches: pull downs; intrinsic fluorescence; and ITC. Moreover, the Munc18c: Sx4 heterodimer was co-purified with an apparent 1∶1 stoichiometry. The binding affinity for Sx4_1-275_-His was ∼104±43 nM for Munc18c expressed from bacteria as compared with 95±15 nM for Munc18c expressed in insect cells [33]. These results indicate that Munc18c expressed in bacteria is correctly folded, and functional in its ability to interact with Sx4.

These findings are encouraging for the use of bacterially expressed Munc18c for future protein-protein interaction and structural studies. The cost of expressing the protein in this way reduces the cost of media and consumables by more than a factor of ten. Also bacterial expression and purification can be completed in 2–3 days rather than 15–17 days for insect cell expressed protein. The typical final yield of purified Munc18c (HMunc18c, HTMunc18c, HLMunc18c or untagged Mun18c) expressed in bacterial cultures was between 1–2 mg per L of cell culture as compared to 3–4 mg per L insect cell culture.

## Supporting Information

Figure S1 **DNA sequences.** **A.** codon optimized gene for mouse full-length Munc18c expression in *E. coli*. **B.** Linker sequence.
(TIF)

Figure S2 **Effect of cell-lysis method on HMunc18c purification.** SDS-PAGE gel showing eluted HMunc18c from IMAC beads after cell lysis by **A.** sonication or **B.** lysozyme treatment.
(TIF)

Figure S3 **Purification of HTMunc18c.** **A.** SDS-PAGE analysis of HTMunc18c purification steps. **B.** TEV cleaved (de-tagged) HTMunc18c obtained by reverse IMAC. **C.** Elution profile of the de-tagged Munc18c from SEC (lanes 1–4).
(TIF)

Figure S4 **Purification of HLMunc18c.** **A.** SDS-PAGE analysis of HLMunc18c IEC fractions. **B.** Elution profile of HLMunc18c from IEC MonoS column.
(TIF)

Figure S5 **Comparison of Munc18c produced from insect or bacterial culture.** **A.** Overlaid SEC chromatograms for HLMunc18c expressed from Sf9 insect cells (dark blue) or *E.coli* (light blue). **B.** Samples injected onto the SEC column in panel A, were assessed by Coomassie-blue stained SDS-PAGE.
(TIF)

Figure S6 **ITC raw data.** The upper panel shows the raw data from a representative experiment for the ITC measured interaction between HMunc18c (in the cell) and Sx4_1-275_-His (in the syringe). The lower panel shows the integrated and normalised data.
(TIF)

Figure S7 **Recombinant Munc18c generated from** ***E. coli*** **expression culture binds to assembled SNARE complex.** Coomassie Blue stained SDS-PAGE gel showing the binding of Munc18c (de-tagged) to pre-formed SNARE ternary complex. The input proteins for this experiment are shown on extreme left. The SNARE complex was formed by mixing solutions of purified Sx4_1-275_-His, SNAP23 and VAMP2 and incubating overnight at 4°C. The SNARE complex was then isolated on TALON Co^2+^ beads. The beads were then incubated for 2 h with de-tagged Munc18c and washed prior to analysis by SDS-PAGE. A sample of the SNARE complex assembled and captured on beads, prior to addition of Munc18c is shown for comparison.
(TIF)
