# Supplementary figures and images for: Milligram Quantities of Homogeneous Recombinant Full-Length Mouse Munc18c from Escherichia coli Cultures
Source: PLoS One. 2013 Dec 31;8(12):e83499. doi: 10.1371/journal.pone.0083499 (PMC3877047; doi:10.1371/journal.pone.0083499)

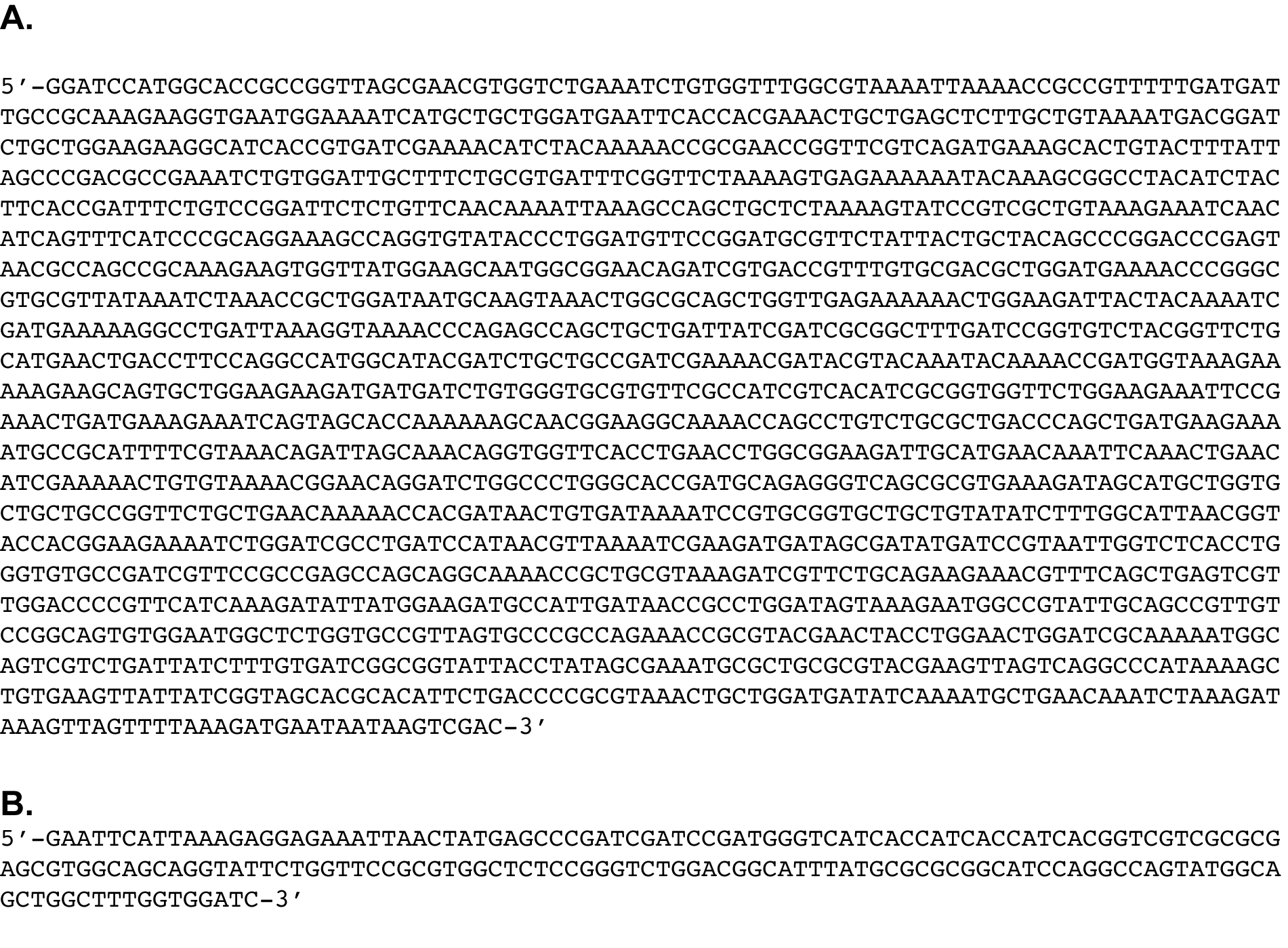

Supplement: Figure S1 — DNA sequences. A. codon optimized gene for mouse full-length Munc18c expression in E. coli. B. Linker sequence. (TIF) [file pone.0083499.s001.tif]

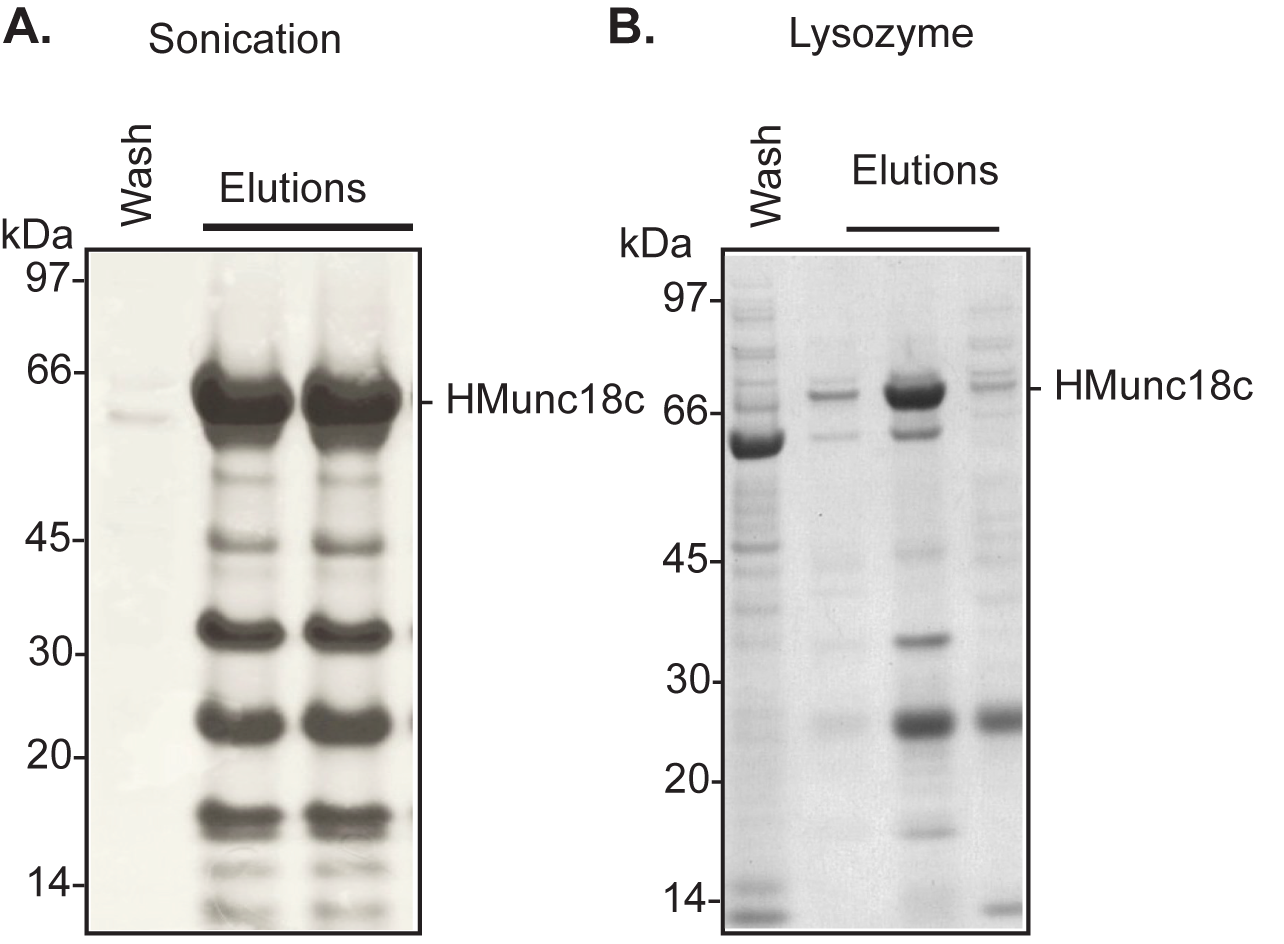

Supplement: Figure S2 — Effect of cell-lysis method on HMunc18c purification. SDS-PAGE gel showing eluted HMunc18c from IMAC beads after cell lysis by A. sonication or B. lysozyme treatment. (TIF) [file pone.0083499.s002.tif]

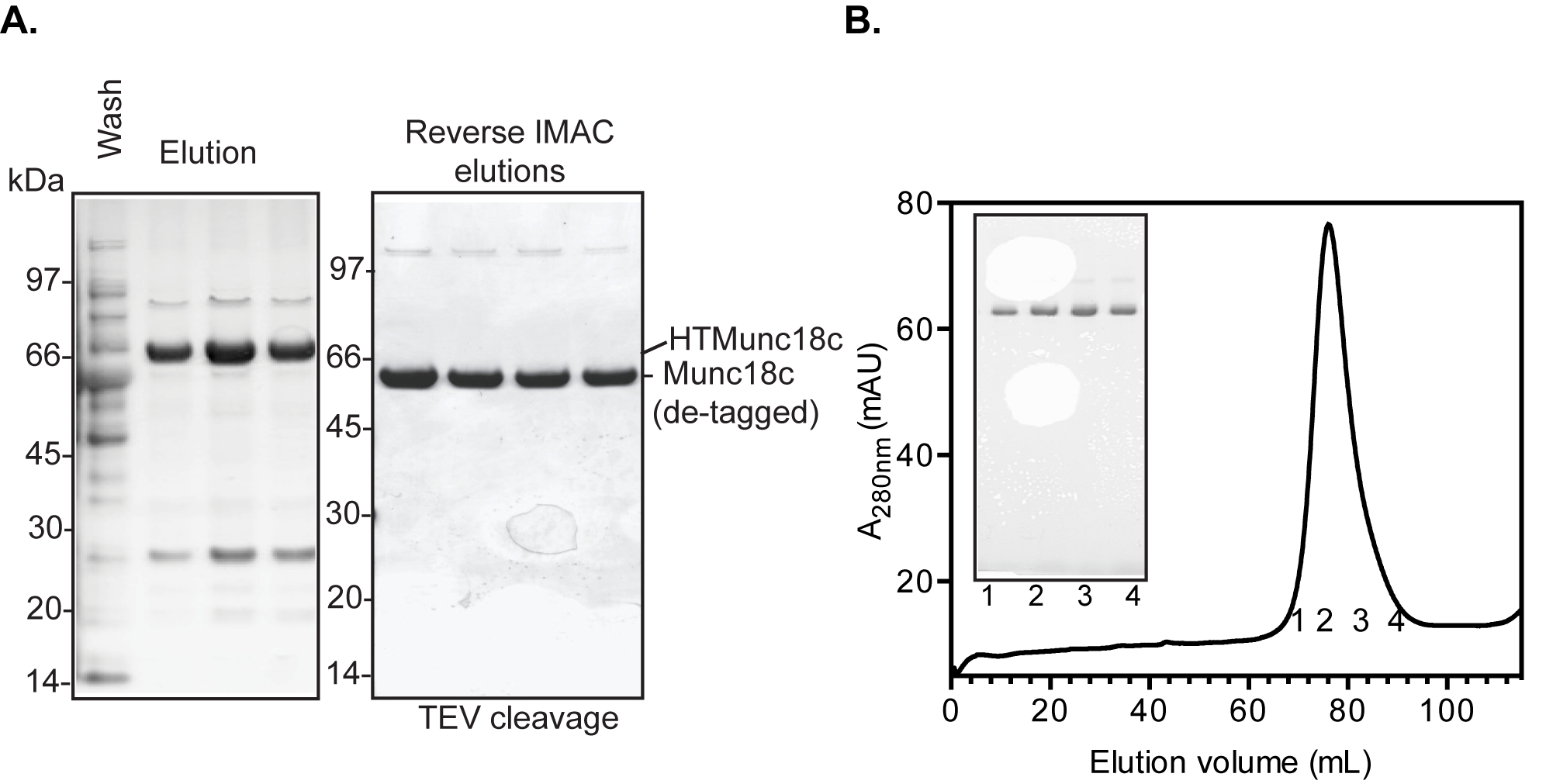

Supplement: Figure S3 — Purification of HTMunc18c. A. SDS-PAGE analysis of HTMunc18c purification steps. B. TEV cleaved (de-tagged) HTMunc18c obtained by reverse IMAC. C. Elution profile of the de-tagged Munc18c from SEC (lanes 1–4). (TIF) [file pone.0083499.s003.tif]

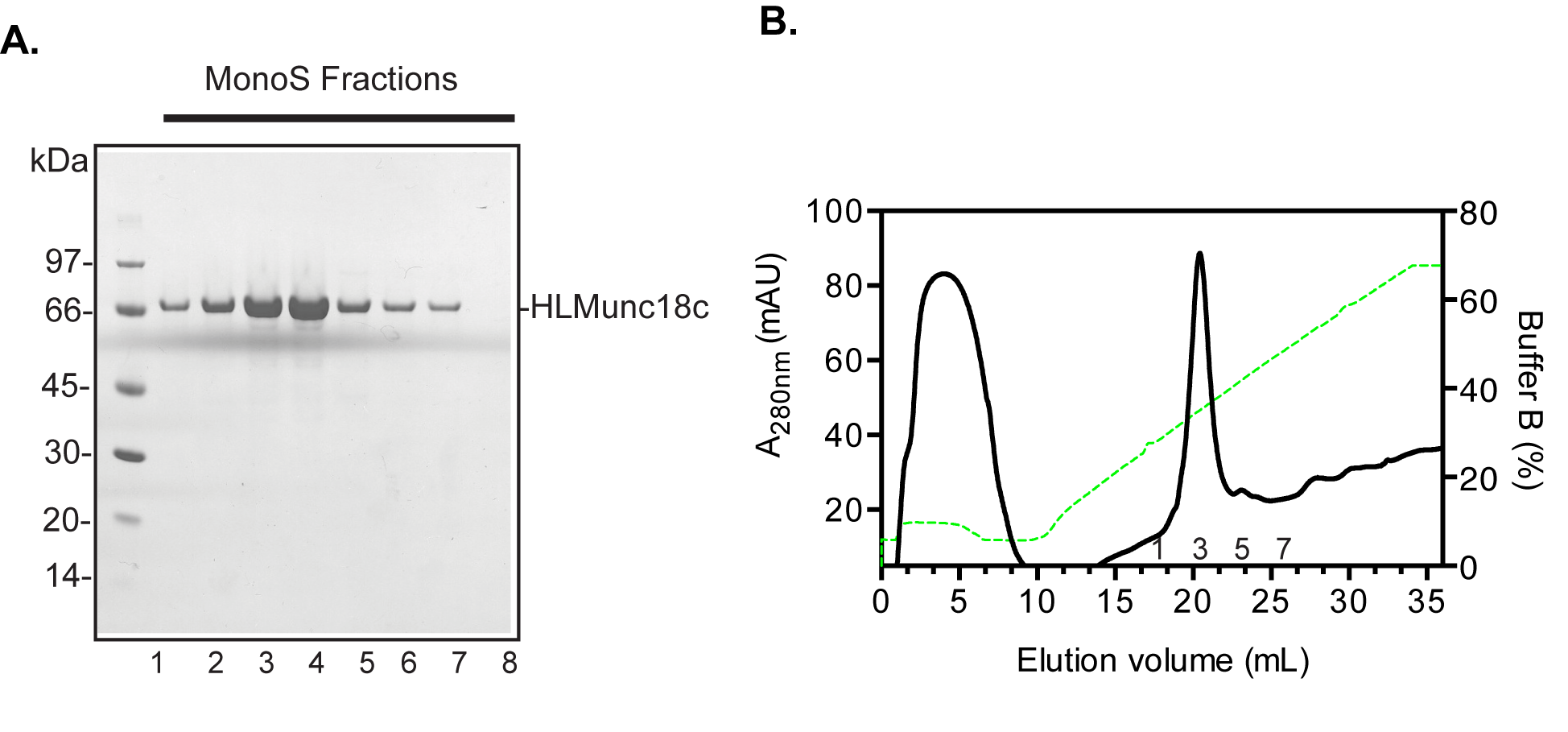

Supplement: Figure S4 — Purification of HLMunc18c. A. SDS-PAGE analysis of HLMunc18c IEC fractions. B. Elution profile of HLMunc18c from IEC MonoS column. (TIF) [file pone.0083499.s004.tif]

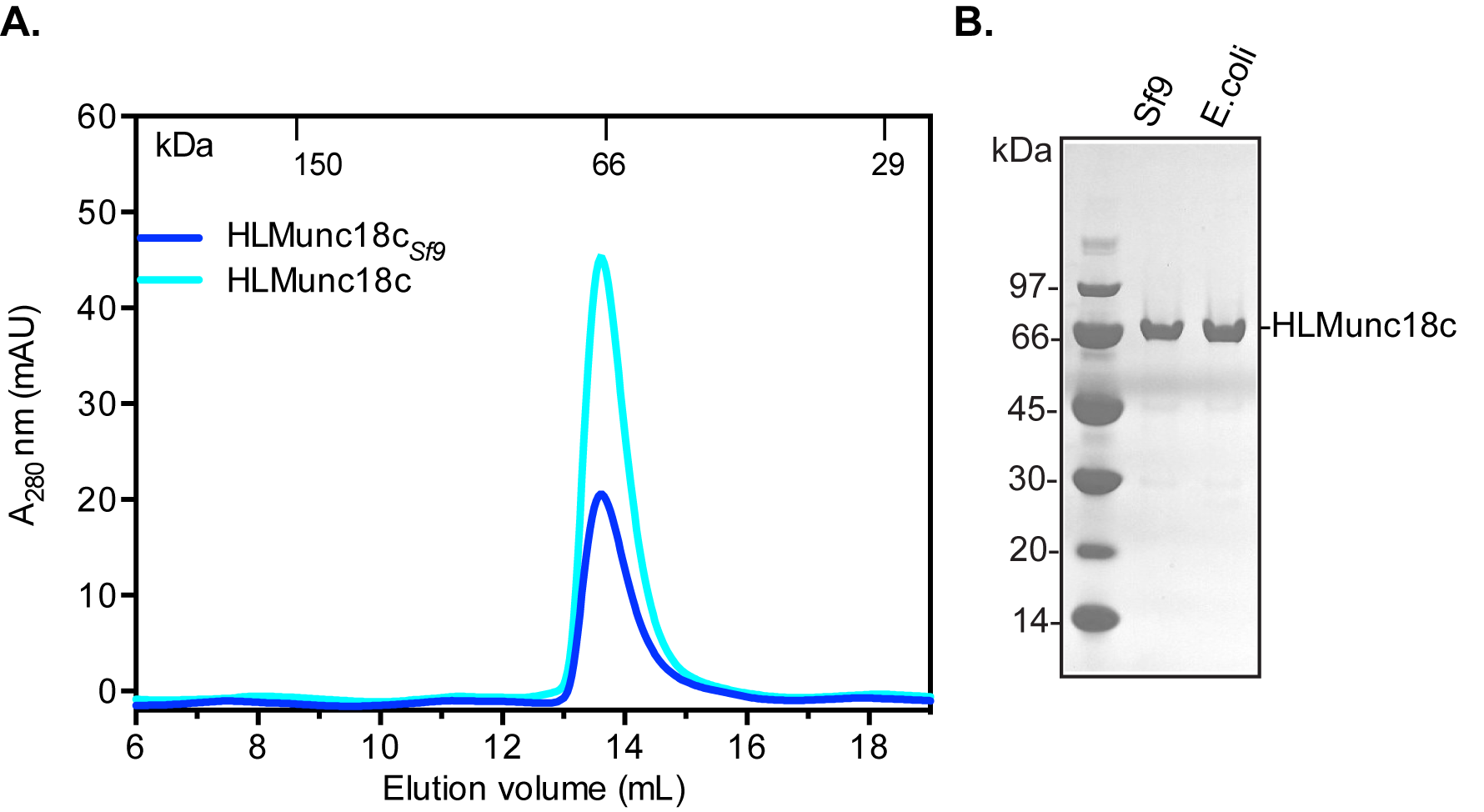

Supplement: Figure S5 — Comparison of Munc18c produced from insect or bacterial culture. A. Overlaid SEC chromatograms for HLMunc18c expressed from Sf9 insect cells (dark blue) or E.coli (light blue). B. Samples injected onto the SEC column in panel A, were assessed by Coomassie-blue stained SDS-PAGE. (TIF) [file pone.0083499.s005.tif]

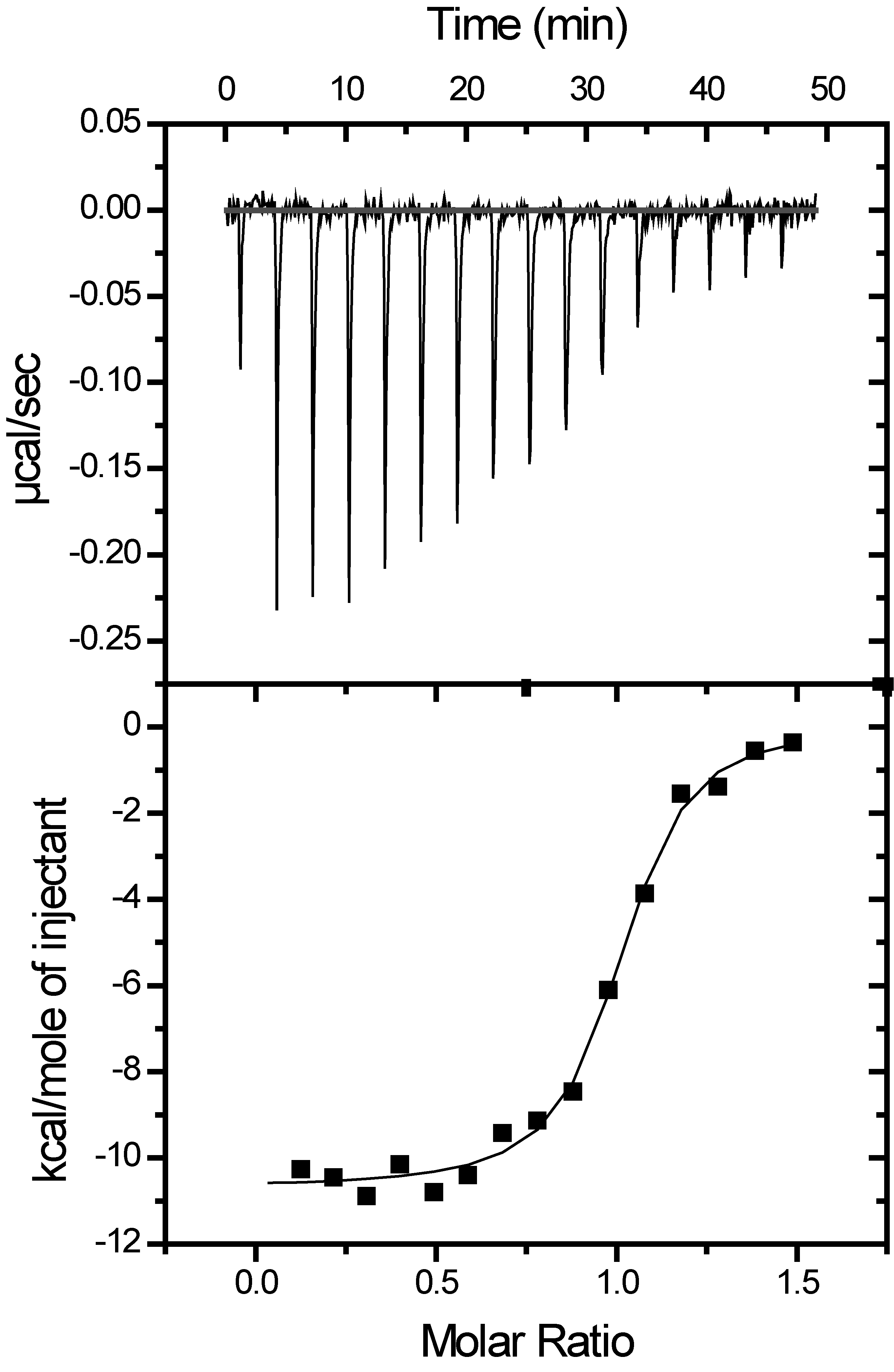

Supplement: Figure S6 — ITC raw data. The upper panel shows the raw data from a representative experiment for the ITC measured interaction between HMunc18c (in the cell) and Sx41-275-His (in the syringe). The lower panel shows the integrated and normalised data. (TIF) [file pone.0083499.s006.tif]

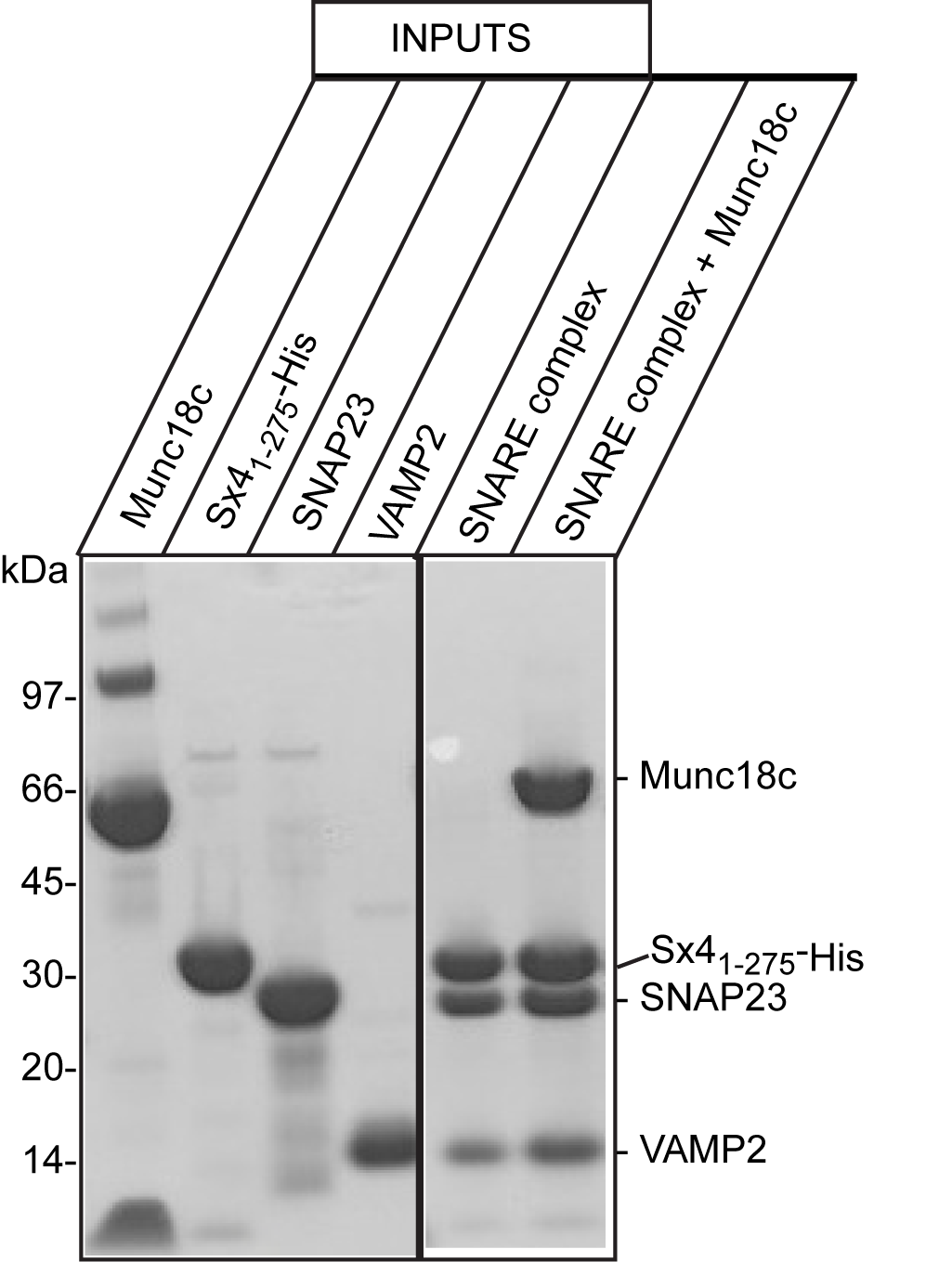

Supplement: Figure S7 — Recombinant Munc18c generated from E. coli expression culture binds to assembled SNARE complex. Coomassie Blue stained SDS-PAGE gel showing the binding of Munc18c (de-tagged) to pre-formed SNARE ternary complex. The input proteins for this experiment are shown on extreme left. The SNARE complex was formed by mixing solutions of purified Sx41-275-His, SNAP23 and VAMP2 and incubating overnight at 4°C. The SNARE complex was then isolated on TALON Co2+ beads. The beads were then incubated for 2 h with de-tagged Munc18c and washed prior to analysis by SDS-PAGE. A sample of the SNARE complex assembled and captured on beads, prior to addition of Munc18c is shown for comparison. (TIF) [file pone.0083499.s007.tif]
